# Breakfast Skipping is Positively Associated With Incidence of Type 2 Diabetes Mellitus: Evidence From the Aichi Workers’ Cohort Study

**DOI:** 10.2188/jea.JE20140109

**Published:** 2015-05-05

**Authors:** Mayu Uemura, Hiroshi Yatsuya, Esayas Haregot Hilawe, Yuanying Li, Chaochen Wang, Chifa Chiang, Rei Otsuka, Hideaki Toyoshima, Koji Tamakoshi, Atsuko Aoyama

**Affiliations:** 1 Department of Public Health and Health Systems, Nagoya University Graduate School of Medicine, Nagoya, Japan; 1 名古屋大学大学院医学系研究科 国際保健医療学・公衆衛生学; 2 Department of Public Health, Fujita Health University School of Medicine, Toyoake, Aichi, Japan; 2 藤田保健衛生大学医学部 公衆衛生学; 3 Section of Longitudinal Study of Aging, National Institute for Longevity Sciences (NILS-LSA), National Center for Geriatrics and Gerontology, Obu, Aichi, Japan; 3 国立長寿医療研究センター 長期縦断疫学研究（NILS-LSA）活用研究室; 4 Anjo Kosei Hospital, Anjo, Aichi, Japan; 4 安城更生病院; 5 Department of Nursing, Nagoya University Graduate School of Medicine, Nagoya, Japan; 5 名古屋大学大学院医学系研究科 看護学専攻

**Keywords:** breakfast, diabetes mellitus, cohort study, Japan

## Abstract

**Background:**

Skipping breakfast has been suspected as a risk factor for type 2 diabetes (T2DM), but the associations are not entirely consistent across ethnicities or sexes, and the issue has not been adequately addressed in the Japanese population.

**Methods:**

We followed 4631 participants (3600 men and 1031 women) in a work-site cohort of participants aged 35–66 years in 2002 through 2011 for T2DM development. Frequency of eating breakfast was self-reported and was subsequently dichotomized to breakfast skippers, who eat breakfast 3–5 times/week or less, and to eaters. Cox proportional hazards models were used to adjust for potential confounding factors, including dietary factors, smoking and other lifestyles, body mass index (BMI), and fasting blood glucose (FBG) at baseline.

**Results:**

During 8.9 years of follow-up, 285 T2DM cases (231 men and 54 women) developed. Compared to participants who reported eating breakfast every day, maximally-adjusted hazard ratios and 95% confidence intervals (CI) of those with the frequency of almost every day and 3–5, 1–2, and 0 days/week were: 1.06 (95% CI, 0.73–1.53), 2.07 (95% CI, 1.20–3.56), 1.37 (95% CI, 0.82–2.29), and 2.12 (95% CI, 1.19–3.76), respectively. In a dichotomized analysis, breakfast skipping was positively associated with T2DM incidence (maximally-adjusted hazard ratio 1.73; 95% CI, 1.24–2.42). The positive associations were found in both men and women, current and non-current smokers, normal weight and overweight (BMI ≥25 kg/m^2^), and normal glycemic status and impaired fasting glycemic status (FBG 110 to <126 mg/dL) individuals at baseline (*P*s for interaction all >0.05).

**Conclusions:**

The present study in middle-aged Japanese men and women suggests that skipping breakfast may increase the risk of T2DM independent of lifestyles and baseline levels of BMI and FBG.

## Abstract (ja)

**背景:**

朝食欠食が2型糖尿病（T2DM）の危険因子であるかもしれないことが示唆されているが、この関連性は民族間や男女によって一致しておらず、また、日本人では十分な検討がされていない。

**方法:**

2002年に35-66歳の某自治体職員4631名（男性3600名、女性1031名）を対象とし、T2DM発症を2011年まで追跡した。朝食の摂取頻度は、自記式アンケートで調査し、週の摂取頻度が3-5回以下の者を朝食欠食者とした。朝食欠食と糖尿病発症との関連は、食事要因や喫煙、その他の生活習慣、ベースライン時のbody mass index（BMI）及び 空腹時血糖（FBG）値を調整したCox比例ハザードモデルで解析した。

**結果:**

8.9年の追跡期間中に、285名（男性231名、女性54名）がT2DMを発症した。朝食を「必ず毎日食べる」と回答した者を基準とした、朝食を「ほぼ毎日食べるが時々食べない」、「週3-5日食べる」、「週1-2日食べる」、「食べない」と回答した者のT2DM発症のハザード比（95%信頼区間）は、その順に1.06 (0.73-1.53)、2.07 (1.20-3.56)、1.37 (0.82-2.29)、2.12 (1.19-3.76) であった。また、朝食欠食者は、朝食摂取者に比べ、T2DM発症のハザード比が有意に高値であった（ハザード比: 1.73、95%信頼区間: 1.24-2.42）。朝食欠食とT2DM発症率の正の関連は、男女、現喫煙者かどうか、過体重（BMI≥25kg/m^2^）の有無、空腹時高血糖（110≤FBG<126mg/dl）の有無によらず、全ての層において認められた（朝食欠食と各層化変数の交互作用項のP>0.05）。

**結論:**

中高年日本人男女を対象とした本研究結果から、朝食欠食が他の生活習慣やベースラインのBMI及びFBG値に独立してT2DM発症リスクを高めることが示唆された。

## INTRODUCTION

Type 2 diabetes mellitus (T2DM) is a major cause of morbidity and mortality globally.^1^ Indeed, the prevalence of diabetes in Japanese aged 40–69 was reported to be as high as 10.2% in male workers and 4.7% in female workers in large-scale companies in 2008–2010 and 15.0% in men and 8.0% in women in the National Health and Nutrition Survey in 2011.^2^^,^^3^ Diabetes was also the 14^th^ highest cause of disability-adjusted life years (DALYs) in Japan in 2010.^4^ It is estimated that diabetes prevalence will remain roughly stable in the next 20 years.^5^

At the same time, skipping breakfast has been suggested to be associated with the incidence of several diseases or conditions, such as obesity,^6^ insulin insensitivity,^7^ cardiovascular diseases,^8^ and T2DM.^9^^–^^11^ However, the associations of breakfast skipping with T2DM are not entirely consistent across different ethnicities or sexes, and the issue has not been adequately addressed in the Japanese population. Furthermore, breakfast skipping is becoming common in Japan, as the 2011 National Health and Nutrition Survey indicated that 14.4% men and 11.1% women start their day without having breakfast.^3^ Since breakfast eating habit is modifiable, examining the possible causal relationship has public health significance in the prevention of T2DM.

Therefore, we designed the present study to examine the association between breakfast skipping and the incidence of T2DM in a large-scale prospective cohort of middle-aged Japanese men and women, and to find out whether the association would be independent of diet and lifestyles, body mass index (BMI), and fasting blood glucose (FBG) levels at baseline.

## METHODS

### Study population

We used data obtained from the Aichi workers’ cohort study. The cohort included 6648 Japanese civil servants in Aichi prefecture, an urban and suburban area located in central Japan. Participants were between 35 and 66 years old at recruitment in 2002. Most workers in the cohort were engaged in clerical work. Police officers, firefighters, and public school teachers were not included in the cohort, but health professionals working in prefectural hospitals were included. The baseline survey conducted in 2002 included a self-administered questionnaire concerning their lifestyle and medical history, as well as a health checkup. Written informed consent was obtained in advance separately for the lifestyle questionnaire and the use of annual health checkup data.

We excluded the following participants: (1) those who did not agree to our use of the annual health checkup results (*n* = 1045); (2) those with missing information for frequency of eating breakfast (*n* = 8), energy intake (*n* = 83), and smoking status (*n* = 48); (3) prevalent cases of diabetes mellitus, defined as self-reported medication use (*n* = 570) or baseline glucose level ≥126 mg/dL (*n* = 128); and (4) those modifying their diet under physicians’ or dieticians’ suggestion (*n* = 135). After these exclusions, 4631 participants (3600 men and 1031 women) were available for the present analysis.

Participants were followed until the end of follow-up (March 31, 2011), censoring, or ascertainment of diabetes, whichever came first. Participants were censored when they retired, except for those who provided their postal address to the researchers. The number of participants who were censored due to retirement was 919. The study protocol was approved by the Ethics Review Committee of Nagoya University School of Medicine.

### Ascertainment of incident T2DM

We ascertained incidence of T2DM by two methods. First, we defined the incidence as the year when FBG level reached ≥126 mg/dL. We arbitrarily set the date of onset as July 1st for the analysis, considering that the checkups were usually carried out from October to December and that T2DM would generally be a chronic state without a definite onset. Second, we utilized data from self-administered questionnaire surveys on medical history, which were conducted in 2004, 2007, and 2011. In the surveys, participants reported medical histories of T2DM and other pre-specified conditions. Those who had T2DM history provided information about the onset (year of diabetes diagnosis) as well as the name and address of their present or past physician. We obtained written consent to access participants’ medical records via their specified physicians. We previously confirmed the accuracy (95%) of self-reports by reviewing the medical records from cases with written consent, and the details of the validation study have been reported elsewhere.^12^ Health checkups were provided annually during their employment. After retirement, participants were followed only by the questionnaire. Health checkup results of retired participants were not systematically collected.

### Frequency of eating breakfast

In this study, we assessed frequency of eating breakfast using a self-administered questionnaire. Breakfast eating frequency was assessed using the following five categories: every day, almost every day with occasional skips, 3–5 days/week, 1–2 days/week, and none. We later reclassified the participants into two groups as breakfast eaters (those who reported their breakfast eating frequency as every day and almost every day with occasional skips) and breakfast skippers (those who classified themselves in the breakfast eating categories of 3–5 days/week, 1–2 days/week, and none). The reproducibility (Spearman’s correlation coefficient) of the breakfast eating frequency question over a 9-month period has been reported to be 0.62.^13^ In addition, 752 out of the present 4631 participants also reported breakfast eating frequency 5 years after the baseline (in 2007). The agreement between these two surveys was still fair (Spearman’s correlation coefficient: 0.55).

### Other dietary factors

The self-administered brief-type dietary history questionnaire (BDHQ) was used for the assessment of diet, including intakes of nutrients, alcohol, and total energy.^14^^,^^15^ Intakes of fish, fruits, vegetables, whole-grain cereals, coffee, sugar-sweetened beverages, and snacks obtained by the BDHQ were adjusted for total energy intake by the nutrient density method.^14^ Eating speed was self-reported in the BDHQ as very fast, relatively fast, medium, relatively slow, and slow. The last two categories were combined in the analysis. In addition to the information obtained by the BDHQ, information about participants’ habit of eating to satiety was also obtained.

### Anthropometric measurements and biochemical analysis

Height was measured to the nearest 0.1 cm with participants standing upright against a stadiometer without shoes. Body weight was measured to the nearest 0.1 kg with the participants in typical indoor clothing. BMI was calculated as weight (kg) divided by the square of height (m). Venous blood samples were drawn after the participants fasted for 8 h or more (or overnight), and serum samples were frozen at −80°C until the biochemical assay. Blood glucose was enzymatically determined by the hexokinase method. Insulin concentration was measured by solid-phase radioimmunoassay (RIABEAD II; Dinabot Co., Ltd., Chiba, Japan).

### Other variables

Smoking status was classified into three categories (current, former, and never). The number of days engaged in leisure-time physical activity for 60 minutes or more was self-reported and classified into two categories: ≥3 days/week or <3 days/week. Work-time physical activity was assessed by the question “Are you engaged in physical labor?” and classified into two categories (yes or no). Work schedule was classified into four categories: with shiftwork including night shifts, with shiftwork but without night shifts, without shiftwork but with night work, and without shiftwork or night work. Sleep duration was classified into two categories: <7 h or ≥7 h. Strength of perceived stress was self-reported by the following four categories: very much, much, ordinary, and little. Family history of diabetes among first-degree relatives was self-reported and used as a dichotomized variable (yes or no) in the analysis.

### Statistical analysis

FBG values were log-transformed to approximately normalize their distribution prior to the analyses and are presented as geometric means and their 95% confidence intervals (CIs). Other continuous variables were summarized as means and standard deviations (SD), while percentages were used for categorical variables. One-way analysis of variance or χ^2^ tests were used, as appropriate, to compare baseline characteristics of breakfast eaters and breakfast skippers.

Multivariable adjusted Cox proportional hazards models were used to estimate hazard ratios (HRs) and 95% CIs of the risk of T2DM according to breakfast eating frequency and in breakfast skippers relative to breakfast eaters. Model 1 was adjusted for age, sex, total energy intake, smoking status, alcohol consumption, leisure-time physical activity, work-time physical activity, family history of diabetes mellitus, eating speed, perceived stress, sleep duration, work schedule, habit of eating to satiety, and intakes of fruits and vegetables, fish, whole-grain cereals, coffee, sugar-sweetened beverages and snacks. In model 2, BMI (continuous) was included in addition to all the variables in model 1. We adjusted for FBG (continuous) in model 3 together with the variables in model 2.

We then performed the above analyses stratified by sex, smoking status (current, never, or former) and baseline values of BMI (<25 kg/m^2^ or ≥25 kg/m^2^) and FBG (<110 mg/dL or ≥110 mg/dL). We also conducted sensitivity analyses by excluding incident cases who were followed up for less than 3 years (*n* = 119) and those who were night shift workers (*n* = 53). Furthermore, we carried out an analysis by censoring all the participants at the age of 60 (retirement age) because not all the retired participants were followed up (*n* of participants who were followed up after retirement = 881).

We also performed an analysis by updating breakfast skipping information with the data obtained after 5 years from baseline when available (approximately 70%) using time-dependent Cox regression model. For those who did not have such information, we carried forward the 2002 frequency.

Another ancillary analysis changing the outcome to impaired fasting glucose (IFG) or diabetes defined as FBG ≥110 mg/dL was performed in an attempt to examine similarity and discrepancy of our findings to a prior study that used such outcome.^16^

All statistical analyses were conducted with IBM SPSS Statistics for Windows, Version 22.0 software (IBM Corp, Armonk, NY, USA). All tests were two-sided, and *P* < 0.05 was considered statistically significant.

## RESULTS

Out of the 4631 participants included in our analysis, 90.4% were breakfast eaters. Compared with breakfast eaters, breakfast skippers seem to have worse lifestyles. For example, breakfast skippers were more likely to be current smokers, consumed more alcohol and sugar-sweetened beverages, and had less intakes of fruits and vegetables (all *P* < 0.05) (Table 1).

**Table 1.** Participants’ demographic, lifestyle, dietary habits, and metabolic risk factor characteristics according to breakfast consumption status at baseline, Aichi, 2002

	Breakfast eaters^a^	Breakfast skippers^b^	*P* value^c^
*n*, %	4188, 90.4	443, 9.6	
Men, %	78.2	73.8	0.04
Age, year	47.8 (7.1)	46.0 (6.8)	<0.001
Body mass index, kg/m^2^	22.9 (2.8)	22.9 (3.0)	0.78
Smoking status, %			
Current	26.7	44.9	<0.001
Former	23.4	14.2	
Never	49.9	40.9	
Leisure-time physical activity, %^d^			
≥3 days/week	75.5	84.2	<0.001
<3 days/week	16.0	9.5	
Work-time physical activity, yes, %	5.3	7.7	<0.01
Family history of diabetes mellitus, yes, %	14.8	17.4	0.14
Fasting blood glucose, mg/dL^e^	92.3 (92.0–92.6)	92.5 (91.5–93.4)	0.74
Perceived stress, %^d^			
Very much	11.0	13.8	0.02
Much	40.1	39.7	
Ordinary	43.8	39.1	
Little	4.9	6.8	
Sleep duration, %^d^			
<7 hours/day	52.9	61.6	<0.001
≥7 hours/day	45.5	35.7	
Work schedule, %^d^			
Without shift work or night shifts	84.6	78.3	<0.001
With shift work but without night shifts	1.9	1.4	
Without shift work but with night shifts	6.8	7.9	
With shift work including night shifts	4.9	11.3	
Total energy intake, kcal/day	1942 (538)	1740 (553)	<0.001
Alcohol consumption, g/day	13.6 (19.2)	18.1 (26.4)	<0.001
Eating speed, %^d^			
Very fast	11.4	12.9	0.67
Relatively fast	35.8	37.9	
Medium	38.5	35.2	
Slow	12.6	12.4	
Satiation eater, %	61.2	59.1	0.24
Fruits and vegetables intake, g/1000 kcal/day	142.3 (67.0)	118.7 (61.2)	<0.001
Fish intake, g/1000 kcal/day	84.1 (41.9)	84.8 (39.4)	0.74
Frequency of whole-grain cereals intake, %			
Always	8.5	5.7	0.10
Sometimes	9.2	8.1	
Rarely	14.9	12.6	
No	67.4	73.6	
Frequency of coffee intake, %^d^			
≥4 cups/day	6.6	10.6	0.02
2–3 cups/day	39.0	39.1	
1 cup/day	24.3	23.7	
<1 cup/day	28.8	25.1	
Frequency of sugar-sweetened beverages intake, %^d^			
≥1 serving/day	5.5	9.3	<0.001
4–6 servings/week	5.3	7.0	
1–3 servings/week	29.9	36.3	
Never or rarely	57.6	45.1	
Snack intake, yes, %	95.3	94.8	0.61

Values are reported as mean (standard deviation) or percentage.

^a^Breakfast eater was defined as those having breakfast eating frequency of ‘every day or almost every day with occasional skips’.

^b^Breakfast skipper was defined as those having breakfast eating frequency of ‘3–5 days/week, 1–2 days/week, or none’.

^c^Obtained from ANOVA and Chi-square test for continuous and categorical variables, respectively.

^d^Proportions in each category do not add up to 100% when there were missing data.

^e^Geometric mean (95% confidence interval).

During a median of 8.9 years of follow-up, 285 cases of T2DM (231 men and 54 women) developed (crude incidence rate: 8.2 per 1000 person-years). Participants who reported eating breakfast 3–5 days/week, 1–2 days/week, and 0 days/week had higher T2DM incidence than those who consumed breakfast every day (model 3 HRs ranging from 1.37 to 2.12) (Table 2). However, the point estimates fluctuated and were not always statistically significant, and there was no apparent positive trend in the T2DM incidence according to the number of days breakfast was skipped. In addition, T2DM incidence of those who skipped breakfast only occasionally was not higher than every day eaters (model 3 HR 1.06). In the subsequent dichotomized analysis, T2DM incidence of breakfast skippers was significantly higher than that of breakfast eaters (crude incidence rate: 13.9/1000 person-years vs 7.5/1000 person-years; model 3 HR 1.73). The positive associations between breakfast skipping and T2DM were similar in both men and women, current and non-current smokers, and normal weight and overweight individuals, as well as those with normal glycemic status and those with impaired fasting glycemic status at baseline (all interaction *P* > 0.05) (Table 3). Furthermore, similar associations were found in sensitivity analyses that excluded incident cases whose follow up periods in the cohort were less than 3 years (model 3 HR 1.94; 95% CI, 1.24–2.98) and those who were night shift workers (model 3 HR 1.91; 95% CI, 1.30–2.80). Also, the association by the analysis that censored all the participants who reached 60 years did not materially differ (model 3 HR 1.71; 95% CI, 1.21–1.49). The analysis updating breakfast skipping information also yielded similar results (model 3 HR 1.66; 95% CI, 1.19–2.32). In another ancillary analysis that used IFG or T2DM as the outcome among participants whose baseline FBG <110 mg/dL (3371 men and 986 women), skipping breakfast was also significantly associated with higher incidence of IFG or T2DM (*n* of incident cases: 707; model 3 HR 1.29; 95% CI, 1.02–1.63).

**Table 2.** Incidence rates and hazard ratios of type 2 diabetes mellitus incidence according to breakfast consumption, Aichi, 2002–2011

	Frequency of eating breakfast				

	Every day	Almost every day with occasional skips	3–5 days/week	1–2 days/week	None
	
*n* of cases/*N*	204/3648	35/540	15/121	17/197	14/125
Crude incidence rate^a^	7.4	8.4	16.6	11.4	15.4
Crude HR (95% CI)	1 (reference)	1.13 (0.79–1.62)	2.25 (1.33–3.80)	1.54 (0.94–2.53)	2.08 (1.21–3.58)
Model 1^b^ HR (95% CI)		1.11 (0.77–1.60)	1.93 (1.12–3.33)	1.51 (0.91–2.51)	1.96 (1.11–3.45)
Model 2^c^ HR (95% CI)		1.11 (0.77–1.60)	1.97 (1.14–3.39)	1.46 (0.87–2.44)	2.09 (1.18–3.68)
Model 3^d^ HR (95% CI)		1.06 (0.73–1.53)	2.07 (1.20–3.56)	1.37 (0.82–2.29)	2.12 (1.19–3.76)

	Breakfast eaters^e^		Breakfast skippers^f^		
	
*n* of cases/*N*	239/4188		46/443		
Crude incidence rate	7.5		13.9		
Crude HR (95% CI)	1 (reference)		1.85 (1.35–2.54)		
Model 1^b^ HR (95% CI)			1.72 (1.23–2.40)		
Model 2^c^ HR (95% CI)			1.74 (1.24–2.43)		
Model 3^d^ HR (95% CI)			1.73 (1.24–2.42)		

CI, confidence interval; HR, hazard ratio; *n*, number; *N*, number of participants.

^a^Crude incidence rate (per 1000 person-years).

^b^Model 1: Adjusted for age, sex, total energy intake, smoking status, alcohol consumption, leisure-time physical activity, work-time physical activity, family history of diabetes mellitus, eating speed, perceived stress, sleep duration, work schedule, satiation eater, fruits and vegetables intake, fish intake, and intake frequencies of whole-grain cereals, coffee, sugar-sweetened beverages, and snacks.

^c^Model 2: Model 1 + body mass index.

^d^Model 3: Model 2 + fasting blood glucose (Log-transformed).

^e^Breakfast eater was defined as those having breakfast eating frequency of ‘every day or almost every day with occasional skips’.

^f^Breakfast skipper was defined as those having breakfast eating frequency of ‘3–5 days/week, 1–2 days/week, or none’.

**Table 3.** Incidence rates and hazard ratios of type 2 diabetes mellitus according to breakfast consumption stratified by sex, body mass index, fasting blood glucose, and smoking status at baseline, Aichi, 2002–2011

	Breakfast eaters^a^	Breakfast skippers^b^	Breakfast eaters^a^	Breakfast skippers^b^
	
Sex	Men		Women	
	
*n* of cases/*N*	197/3273	34/327	42/915	12/116
Crude incidence rate^c^	7.8	13.8	6.4	14.4
Model 3^d^ HR (95% CI)	1 (reference)	1.54 (1.05–2.28)	1 (reference)	2.29 (1.05–5.02)

Smoking status	Current smoker		Never or former smoker	
	
*n* of cases/*N*	85/1118	22/199	154/3070	24/244
Crude incidence rate	10.2	14.9	6.6	13.1
Model 3^d^ HR (95% CI)	1 (reference)	1.41 (0.85–2.33)	1 (reference)	2.17 (1.36–3.46)

Body mass index	<25 kg/m^2^		≥25 kg/m^2^	
	
*n* of cases/*N*	148/3298	28/332	91/890	18/111
Crude incidence rate	5.9	11.0	13.6	23.6
Model 3^d^ HR (95% CI)	1 (reference)	1.67 (1.08–2.58)	1 (reference)	1.98 (1.13–3.47)

Fasting blood glucose	<110 mg/dL		≥110 mg/dL	
	
*n* of cases/*N*	177/3943	36/414	62/245	10/29
Crude incidence rate	5.9	11.5	41.4	55.1
Model 3^d^ HR (95% CI)	1 (reference)	1.80 (1.23–2.64)	1 (reference)	1.62 (0.75–3.52)

CI, confidence interval; HR, hazard ratio; *n*, number; *N*, number of participants.

^a^Breakfast eater was defined as those having breakfast eating frequency of ‘every day or almost every day with occasional skips’.

^b^Breakfast skipper was defined as those having breakfast eating frequency of ‘3–5 days/week, 1–2 days/week, or none’.

^c^Crude incidence rate (per 1000 person-years).

^d^Model 3 was adjusted for age, sex (if appropriate), total energy intake, smoking status (if appropriate), alcohol consumption, leisure-time physical activity, work-time physical activity, family history of diabetes mellitus, eating speed, perceived stress, sleep duration, work schedule, satiation eater, fruits and vegetables intake, fish intake, and intake frequencies of whole-grain cereals, coffee, sugar-sweetened beverages, and snacks, as well as body mass index (continuous) and fasting blood glucose (Log-transformed).

## DISCUSSION

In the present study, we found that breakfast skipping was positively associated with T2DM incidence in middle-aged Japanese men and women, after adjustment for a number of potential confounding variables, including baseline BMI and FBG levels. We confirmed the associations in both men and women, and in individuals with or without overweight or IFG at baseline. Although the association in current smokers was not statistically significant, formal test of interaction did not suggest any statistically significant difference in the association between current and non-current smokers. The positive association between breakfast skipping and T2DM observed in our study is in line with previous studies conducted in the United States.^9^^–^^11^ We extended the finding to a Japanese sample and confirmed the associations in several subgroups.

The positive association between breakfast skipping and the risk of T2DM shown in our study is also roughly in line with a previous study in Japan,^16^ although the study only found the association in women, did not adjust for important lifestyle variables or perform detailed stratified analyses, and used IFG as the outcome. Our ancillary analysis that employed IFG or T2DM as the outcome found a weaker but similar association as the original analysis.

In the present study, we did not find a dose-response association between breakfast eating (skipping) frequency and T2DM incidence. This is not consistent with a finding from the CARDIA Study (average baseline age: 32 years), which reported a stepwise inverse association between breakfast eating frequency and T2DM incidence in white men and women and in black men but not black women. The reason for the discrepancy is not clear. Other studies conducted in the United States did not specifically address the issue of dose-response. Also, caution is required in interpreting results, since the definition of skipping breakfast differs by studies.^10^^,^^11^

Since previous studies in Japan reported associations between breakfast skipping and higher BMI^6^^,^^17^ as well as regular smoking, we attempted to examine the association precisely by stratifying the analyses by these variables, and we found that the associations were similar across the strata. Nonetheless, the relatively stronger effect estimates observed among those in the higher baseline BMI categories may somehow corroborate the belief that BMI could influence participants’ dietary habits, including breakfast eating behavior, and hence may modify its association with T2DM.^6^^,^^17^ However, whether or not this was really due to the influence of participants’ prior knowledge of their BMI and FBG needs further investigation.

We speculated a few mechanisms by which breakfast skipping could potentially cause T2DM. First, it has been reported that after-lunch postprandial glucose and insulin levels were significantly higher in participants who skipped breakfast than those who consumed breakfast.^18^ Similarly, omitting breakfast has been reported to impair postprandial insulin sensitivity.^7^ Since the level of 1,5-anhydroglucitol, an indicator for short-term hyperglycemia and glycemic excursions, was significantly associated with the development of diabetes independent of HbA1c levels, metabolic alterations induced by breakfast skipping may predispose individuals to diabetes.^19^ Second, skipping breakfast could mean having infrequent larger meals. Total energy intakes in the present study were 1942 and 1740 kcal/day in breakfast eaters and breakfast skippers, respectively. We could guess that breakfast skippers had more energy intake per serving, which means that they had larger serving sizes than breakfast eaters. This may also be associated with future diabetes incidence through greater postprandial glucose and insulin responses. The relationship between breakfast skipping and T2DM observed in our study could also be due to residual confounding of other lifestyles.^17^ Although we have adjusted for a number of lifestyles, breakfast skippers may have other lifestyle and behavior characteristics that may cause T2DM.

Our study has several other limitations. First, although the BDHQ had fair reproducibility over 9 months as well as 5 years, breakfast consumption was self-reported and subject to a subjective interpretation of what constitutes a breakfast and duration of fasting before the meal. However, the possible information bias as a result would be non-differential and is not expected to influence our findings. Indeed, no material difference in our finding was seen after excluding night shift workers who may have the habit of taking early morning snacks.^20^^,^^21^ Second, there was no information on the nutrient composition of the breakfast consumed, and we could not assess the effect of quality of breakfast on the association between breakfast consumption and T2DM. Since breakfast cereal intake was inversely associated with T2DM incidence,^22^ the association might have been different if we had the information. Third, we ascertained T2DM incidence by a single identification of diabetic FBG level. Although this definition is commonly employed in epidemiologic studies, additional measurement of HbA1c or oral glucose tolerance test results should ideally be used. We also utilized self-reports for T2DM ascertainment, which would have high specificity but relatively low sensitivity.^23^ Finally, the observational nature of the present study would prevent any definitive statement about causality. Although our study participants were relatively homogeneous middle-aged Japanese civil servants with similar demographic, socioeconomic, and environmental backgrounds, there may still be unknown or uncontrolled confounding. While a long-term randomized controlled trial would be difficult to conduct, well-designed cohort studies in other settings may be useful, since findings on the issue are still scarce and have not always been consistent.

In summary, our findings indicate that skipping breakfast increased the risk of T2DM in middle-aged Japanese workers, and the association was independent of several dietary and lifestyle factors, as well as baseline levels of BMI and FBG. Public health messages promoting the benefits of eating breakfast could be distributed in civil service institutions in Japan and the public at large.

## ONLINE ONLY MATERIAL

Abstract in Japanese.

## References

[1] Danaei G, Finucane MM, Lu Y, Singh GM, Cowan MJ, Paciorek CJ, . National, regional, and global trends in fasting plasma glucose and diabetes prevalence since 1980: systematic analysis of health examination surveys and epidemiological studies with 370 country-years and 2.7 million participants. Lancet. 2011;378:31–40. 10.1016/S0140-6736(11)60679-X 21705069

[2] Uehara A, Kurotani K, Kochi T, Kuwahara K, Eguchi M, Imai T, . Prevalence of diabetes and pre-diabetes among workers: Japan Epidemiology Collaboration on Occupational Health Study. Diabetes Res Clin Pract. 2014;106:118–27. 10.1016/j.diabres.2014.07.013 25112921

[3] Ministry of Health, Labour and Welfare. The National Health and Nutrition Survey in Japan, 2011. Accessed Sep 25, 2014, at: http://www.mhlw.go.jp/bunya/kenkou/eiyou/h23-houkoku.html. 2013 (in Japanese).

[4] Institute for Health Metrics and Evaluation UoW. GBD Country Profile: Japan [Internet]. March 5, 2013 [cited 2014 September 25]. Available from: http://www.healthdata.org/sites/default/files/files/country_profiles/GBD/ihme_gbd_country_report_japan.pdf.

[5] Wild S, Roglic G, Green A, Sicree R, King H. Global prevalence of diabetes: estimates for the year 2000 and projections for 2030. Diabetes Care. 2004;27:1047–53. 10.2337/diacare.27.5.1047 15111519

[6] Timlin MT, Pereira MA. Breakfast frequency and quality in the etiology of adult obesity and chronic diseases. Nutr Rev. 2007;65:268–81. 10.1111/j.1753-4887.2007.tb00304.x 17605303

[7] Farshchi HR, Taylor MA, Macdonald IA. Deleterious effects of omitting breakfast on insulin sensitivity and fasting lipid profiles in healthy lean women. Am J Clin Nutr. 2005;81:388–96. 15699226 10.1093/ajcn.81.2.388

[8] Cahill LE, Chiuve SE, Mekary RA, Jensen MK, Flint AJ, Hu FB, . Prospective study of breakfast eating and incident coronary heart disease in a cohort of male US health professionals. Circulation. 2013;128:337–43. 10.1161/CIRCULATIONAHA.113.001474 23877060 PMC3797523

[9] Odegaard AO, Jacobs DR Jr, Steffen LM, Van Horn L, Ludwig DS, Pereira MA. Breakfast frequency and development of metabolic risk. Diabetes Care. 2013;36:3100–6. 10.2337/dc13-0316 23775814 PMC3781522

[10] Mekary RA, Giovannucci E, Willett WC, van Dam RM, Hu FB. Eating patterns and type 2 diabetes risk in men: breakfast omission, eating frequency, and snacking. Am J Clin Nutr. 2012;95:1182–9. 10.3945/ajcn.111.028209 22456660 PMC3325839

[11] Mekary RA, Giovannucci E, Cahill L, Willett WC, van Dam RM, Hu FB. Eating patterns and type 2 diabetes risk in older women: breakfast consumption and eating frequency. Am J Clin Nutr. 2013;98:436–43. 10.3945/ajcn.112.057521 23761483 PMC3712552

[12] Wada K, Yatsuya H, Ouyang P, Otsuka R, Mitsuhashi H, Takefuji S, . Self-reported medical history was generally accurate among Japanese workplace population. J Clin Epidemiol. 2009;62:306–13. 10.1016/j.jclinepi.2008.04.006 18774692

[13] Yatsuya H, Ohwaki A, Tamakoshi K, Wakai K, Koide K, Otsuka R, . Reproducibility and validity of a simple checklist-type questionnaire for food intake and dietary behavior. J Epidemiol. 2003;13:235–45. 10.2188/jea.13.235 14604218 PMC9691396

[14] Kobayashi S, Murakami K, Sasaki S, Okubo H, Hirota N, Notsu A, . Comparison of relative validity of food group intakes estimated by comprehensive and brief-type self-administered diet history questionnaires against 16 d dietary records in Japanese adults. Public Health Nutr. 2011;14:1200–11. 10.1017/S1368980011000504 21477414

[15] Kobayashi S, Honda S, Murakami K, Sasaki S, Okubo H, Hirota N, . Both comprehensive and brief self-administered diet history questionnaires satisfactorily rank nutrient intakes in Japanese adults. J Epidemiol. 2012;22:151–9. 10.2188/jea.JE20110075 22343326 PMC3798594

[16] Sugimori H, Miyakawa M, Yoshida K, Izuno T, Takahashi E, Tanaka C, . Health risk assessment for diabetes mellitus based on longitudinal analysis of MHTS database. J Med Syst. 1998;22:27–32. 10.1023/A:1022650305109 9554107

[17] Keski-Rahkonen A, Kaprio J, Rissanen A, Virkkunen M, Rose RJ. Breakfast skipping and health-compromising behaviors in adolescents and adults. Eur J Clin Nutr. 2003;57:842–53. 10.1038/sj.ejcn.1601618 12821884

[18] Nakamura Y, Sanematsu K, Ohta R, Shirosaki S, Koyano K, Nonaka K, . Diurnal variation of human sweet taste recognition thresholds is correlated with plasma leptin levels. Diabetes. 2008;57:2661–5. 10.2337/db07-1103 18633111 PMC2551675

[19] Selvin E, Rawlings AM, Grams M, Klein R, Steffes M, Coresh J. Association of 1,5-Anhydroglucitol with Diabetes and Microvascular Conditions. Clin Chem. 2014;60(11):1409–18. 10.1373/clinchem.2014.229427 25200356 PMC4215646

[20] Esquirol Y, Bongard V, Mabile L, Jonnier B, Soulat JM, Perret B. Shift work and metabolic syndrome: respective impacts of job strain, physical activity, and dietary rhythms. Chronobiol Int. 2009;26:544–59. 10.1080/07420520902821176 19360495

[21] Yoshizaki T, Tada Y, Kodama T, Mori K, Kokubo Y, Hida A, Influence of shiftwork on association between body mass index and lifestyle or dietary habits in female nurses and caregivers. Nippon Eiyo Shokuryo Gakkaishi. 2010;63:161–7 (in Japanese) 10.4327/jsnfs.63.161

[22] Kochar J, Djoussé L, Gaziano JM. Breakfast cereals and risk of type 2 diabetes in the Physicians’ Health Study I. Obesity (Silver Spring). 2007;15:3039–44. 10.1038/oby.2007.362 18198313

[23] Goto A, Morita A, Goto M, Sasaki S, Miyachi M, Aiba N, . Validity of diabetes self-reports in the Saku diabetes study. J Epidemiol. 2013;23:295–300. 10.2188/jea.JE20120221 23774288 PMC3709549

