# Supplementary material for: Breakfast Skipping is Positively Associated With Incidence of Type 2 Diabetes Mellitus: Evidence From the Aichi Workers’ Cohort Study
Source: J Epidemiol. 2015 May 5;25(5):351–8. doi: 10.2188/jea.JE20140109 (PMC4411234; doi:10.2188/jea.JE20140109)
Supplement: Abstract in Japanese. [file je-25-351-s001.pdf]

朝食欠食は2 型糖尿病発症と関連する－愛知職域コホート研究の結果から

上村 真由[1]、八谷 寛[1,2]、イサヤス ハレゴットゥ ヒラウェ[1]、李 媛英[2]、
王 超辰[1]、江 啓発[1]、大塚 礼[3]、豊嶋 英明[4]、玉腰 浩司[5]、青山 温子[1]

[1] 名古屋大学大学院医学系研究科 国際保健医療学・公衆衛生学
[2] 藤田保健衛生大学医学部 公衆衛生学
[3] 国立長寿医療研究センター 長期縦断疫学研究（NILS-LSA）活用研究室
[4] 安城更生病院
[5] 名古屋大学大学院医学系研究科 看護学専攻

背景: 朝食欠食が2型糖尿病（T2DM）の危険因子であるかもしれないことが示唆されているが、この関連性は民族間や男女によって一致しておらず、また、日本人では十分な検討がされていない。

方法: 2002年に35-66歳の某自治体職員4631名（男性3600名、女性1031名）を対象とし、T2DM発症を2011年まで追跡した。朝食の摂取頻度は、自記式アンケートで調査し、週の摂取頻度が3-5回以下の者を朝食欠食者とした。朝食欠食と糖尿病発症との関連は、食事要因や喫煙、その他の生活習慣、ベースライン時のbody mass index（BMI）及び空腹時血糖（FBG）値を調整したCox比例ハザードモデルで解析した。

結果: 8.9年の追跡期間中に、285名（男性231名、女性54名）がT2DMを発症した。朝食を「必ず毎日食べる」と回答した者を基準とした、朝食を「ほぼ毎日食べるが時々食べない」、「週3-5日食べる」、「週1-2日食べる」、「食べない」と回答した者のT2DM発症のハザード比（95%信頼区間）は、その順に1.06（0.73-1.53)、2.07（1.20-3.56)、1.37（0.82-2.29)、2.12（1.19-3.76）であった。また、朝食欠食者は、朝食摂取者に比べ、T2DM発症のハザード比が有意に高値であった（ハザード比: 1.73、95%信頼区間: 1.24-2.42）。朝食欠食とT2DM発症率の正の関連は、男女、現喫煙者かどうか、過体重（BMI ≥25kg/m^2）の有無、空腹時高血糖（110≤FBG<126mg/dl）の有無によらず、全ての層において認められた（朝食欠食と各層化変数の交互作用項のP>0.05）。

結論: 中高年日本人男女を対象とした本研究結果から、朝食欠食が他の生活習慣やベースラインのBMI及びFBG値に独立してT2DM発症リスクを高めることが示唆された。

キーワード: 朝食、糖尿病、コホート研究、日本
